# Tobacco use and its association with HPV infection in normal uterine cervix: A study from a Sustainable Development Goals perspective

**DOI:** 10.18332/tid/140093

**Published:** 2021-08-06

**Authors:** Tofan W. Utami, Fitriyadi Kusuma, Hariyono Winarto, Tricia D. Anggraeni, Alexander A. W. Peters, Vivian Spaans, Inas R Humairah, Vani Ardiani, Ahmad R. H. Utomo, Muhamad S. Dahlan

**Affiliations:** 1 Division of Gynecologic Oncology, Department of Obstetrics and Gynecology, Universitas Indonesia, Central Jakarta, Indonesia; 2 Department of Gynecology, Leiden University Medical Center, Leiden, Netherlands; 3 Department of Pathology, Leiden University Medical Center, Leiden, Netherlands; 4 Department of Research and Development, Dharmais Cancer Hospital, West Jakarta, Indonesia; 5 Clinical Epidemiology Department, Epidemiologi Indonesia, East Jakarta, Indonesia

**Keywords:** HPV infection, tobacco use, SDGs, negative VIA, normal uterine cervix

## Abstract

**INTRODUCTION:**

To achieve the Sustainable Development Goals (SDGs) target 2030, the United Nations (UN) endorsed tobacco use reduction, which is essential in decreasing unnecessary deaths caused by tobacco-induced disease. This study investigates the association between tobacco use and Human Papillomavirus (HPV) infection in clinically normal uterine cervix populations from the SDGs perspective.

**METHODS:**

This study is a 7-year cross-sectional study of a clinically normal uterine cervix population, based on negative Visual Inspection of Acetic Acid (VIA). Subjects were recruited consecutively from the medical records of several public and private health providers in Jakarta. The Statistical Product and Service Solutions (SPSS) for Windows version 20.0 were used to analyze the data.

**RESULTS:**

A total of 1397 negative VIA subjects were collected, consisting of 4.9% (69/1397) tobacco users, and 95.1% (1328/1397) non-users. HPV-DNA positive were 4.3% (3/69) in the tobacco user group and 3.7% (49/1328) in the non-user group. Statistical analysis showed unadjusted OR was 1.19 (95% CI: 0.36–3.91, p=0.778) and adjusted OR was 1.18 (95% CI: 0.36–3.89, p=0.786). High-risk HPV (hrHPV) infections of tobacco and non-tobacco users’ groups were found in 2/3 and 27/49 (55.1%), respectively.

**CONCLUSIONS:**

This study showed an insignificant statistical association between tobacco use and HPV infection in normal uterine cervix. However, the proportion of hrHPV infection was higher in tobacco users than non-users. From the SDGs perspective, cervical cancer is closely related to tobacco use and poverty. Further study is needed to support this result and evaluate other co-factor role-related cervical cancer history to achieve SDGs in 2030.

## INTRODUCTION

International Agency for Research on Cancer (IARC), in 2004, listed cervical cancer among those causally related to smoking^1^. A collaborative reanalysis of 12 studies also supported that current tobacco smoking was associated with an increased risk of squamous cell carcinoma (SCC), but not adenocarcinoma (AC)^2-5^. Risk factors associated with an increased incidence of severe dysplasia include sexual intercourse within five years of menarche (OR 3.32–4.09)^6^, lifetime smoking exposure (OR=7.2)^7^, and multiple sexual partners (OR=2.1) with four or more sexual partners^8^.

Understanding the history of HPV infection in the uterine cervix enables mapping out strategies to prevent cervical cancer^9^. Increasing the number of HPV infections has been associated with tobacco use. Tobacco can affect the replication of HPV-DNA and accelerate the HPV life cycle. As a consequence, it can increase the ability of the virus to damage the cervical epithelium. Some cigarette components and their metabolites such as Benzo[a]pyrene, nicotine, and nicotine derived nitrosamines 4-(methylnitrosamino)-1-(3-pyridyl)-1-butanone were found to damage the local defence mechanism in the cervical epithelium^10^. Women who smoke tend to become infected with HPV with a 1.7 odds ratio (95% CI: 1.2–2.2)^11^. An estimated 19.33% of the global population (133 countries)^12^, 29% of the South-East Asia Region (SEAR) population^13^, and 24.3% of the Indonesian population smoke^14^. Although the most active smokers are men, women also contribute, especially as passive smokers, resulting in the same negative effects of smoking^15^.

In 2018, the proportion of women smokers worldwide was at 6.1%^16^, compared to 14.1% in America^17^. The proportion of female smokers in the same year in SEAR and Indonesia were 10.8% and 4.8%, respectively^14^. In Indonesia, smoking cigarettes, the most popular tobacco usage, starts from the age of 12–13 years and higher in the rural than the urban areas^18^. Nevertheless, an increasing number of sexual partners of smoking women might be a potent risk factor^10^. In America, 42% of female teenagers had had sexual intercourse by the age of 15–19 years^19^. On the other hand, in Indonesia, based on a study by Rizkianti et al.^20^, most women delayed (>18 years) sexual onset (98.8%) while only 1.2% of 3943 samples had early (≤18 years) sexual onset.

The SDG 3 is sustained by WHO Framework Convention on Tobacco Control (FTCT) with some ambitious targets including reduction of the prevalence of current tobacco use, reduction of premature mortality from non-communicable diseases and the prevalence of several smokingrelated diseases, and strengthening of research capacity, particularly developing countries^21^. Tobacco use prevalence is highest in low-income countries, while tobacco use contributes to increasing poverty^21^. Consequently, tobacco control, smoking cessation and poverty elimination are becoming some of the United Nation’s main concerns regarding the Sustainable Development Goals (SDGs). Smoking habits and poverty are part of a vicious circle that is difficult to break. This is an important study because there are no data in Indonesia. This study aimed to discover the association between tobacco use and HPV infection in normal uterine cervix from the SDGs perspective.

## METHODS

**Figure 1:**
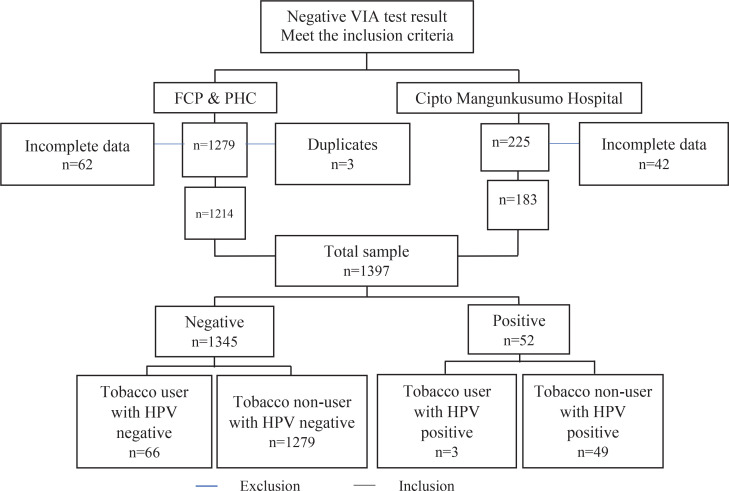
Subject recruitment flowchart

The cross-sectional study data were collected from the medical records for approximately seven years (January 2012 to July 2018). Subjects were women of reproductive age who were admitted to Primary Health Care (PHC) appointed in ‘See and Treat’ Female Cancer Program (FCP) Jakarta, in outpatients of the private clinic of the Obstetrics and Gynecology Department of Dr. Cipto Mangunkusumo General Hospital (Figure 1).

After approval by University of Indonesia Review Board, the data were collected by consecutive sampling and presented with a percentage table from each risk factor. The inclusion criteria were married or sexually active and a negative VIA test. The exclusion criteria were diagnosed with cervical cancer or a pre-cancerous lesion, pregnancy, or genital infection when the study was conducted.

The authors collected data from the medical records of the patients involved in the FCP program and other study sites. Tobacco users were defined as patients who had been ever and/or active cigarette smokers in their lifetime. HPV-DNA status was defined as a positive or negative HPV-DNA test result (PCR SPF10-DEIA-LiPA25 and hybridization for detecting and genotyping HPV-DNA). The samples were collected from cervicovaginal mucus taken before the VIA test. HPV types 16, 18, 31, 39, 51, 52, 53, 56, 58, 68, 69, 73 and 74 were classified as high-risk or probably high-risk types, while HPV 6, 11, 44, 54, 66 and 71 were classified as low-risk. Age was considered as the confounding factor in this study.

The data were processed using Statistical Product and Service Solutions (SPSS) for Windows version 20.0. Association between smoking status and HPV infection was analyzed using chi-squared, and the confounding variable was analyzed using logistic regression.

## RESULTS

A total of 1504 subjects (1279 subjects from FCP and PHC medical records, and 225 subjects from Cipto Mangunkusumo Hospital’s medical records) for seven years met the inclusion criteria. Among these subjects, 62 subjects from FCP and PHC, and 42 subjects from Cipto Mangunkusumo Hospital, were excluded due to incomplete data on age, while three subjects were excluded due to duplicate patient data. Thus, 1397 subjects (1214 subjects from FCP and PHC, and 183 subjects from Cipto Mangunkusumo Hospital) with complete data were processed. Of 1345 subjects with a negative HPV-DNA test, 66 were tobacco users, while 1279 were non-users. Of 52 subjects with a positive HPV-DNA test, 3 were tobacco users, while 49 were non-users.

The subject characteristics are described in Table 1. The median age was 41 years (range: 19–84), with an almost even distribution in each age category. Most of the study subjects (89.2%) were married once in their lifetime, followed by married more than once (5.5%), and widows (4.8%), with a median marital age of 22 years.

**Table 1:** Demographic characteristics (N=1397)

*Characteristics*	*n (%)*
**Age** (years), median (range)	41 (19–84)
**Age** (years), n (%)	
≤40	681 (48.7)
>40	716 (51.3)
**Marital status^a^**	
Married once	1246 (89.2)
Married more than once	77 (5.5)
Widow	67 (4.8)
Marital age (years), median (range)^b^	22 (13–48)
**Tobacco use status**	
User	69 (4.9)
Non-user	1328 (95.1)

a n=1390, as many as 7 subjects missing data.

b n=1391, as many as 6 subjects missing data.

The prevalence of HPV infection of the negative VIA population in this study was 52 subjects (3.7%). Based on tobacco user status, a positive HPV-DNA result was found in 3 (4.3%) tobacco users and 49 (3.7%) non-users. Data analysis showed that the odds ratio of tobacco use was 1.19 (95% CI: 0.36–3.91, p=0.778), and adjusted OR was 1.18 (95% CI: 0.36–3.89, p=0.786) controlling for age, as described in Table 2. The HPV genotypes of the positive subjects are described in Table 3. The infections of hrHPV for tobacco and non-tobacco users were found in 2/3 and 27/49 (55.1%), respectively.

**Table 2:** Association between tobacco use and HPV-DNA in normal uterine cervix

*Tobacco use status*	*HPV-DNA n (%)*		Unadjusted*			Adjusted**		
	Positive	Negative	p	OR	95% CI	p	AOR	95% CI
Users	3 (4.3)	66 (95.7)	0.778	1.19	0.36–3.91	0.786	1.18	0.36–3.89
Non-users	49 (3.7)	1279 (96.3)						

* Chi-squared.

** Logistic regression for etiology; the confounding controlled variable was age. AOR: adjusted odds ratio.

**Table 3:** HPV genotypes distribution

*Subject group*	*Risk stratification*	*HPV genotype*	*n*
**Tobacco users**			
	**hrHPV**		
		HPV 16	1
		HPV 18, 11, 39	1
	**Unknown HPV type**		
		HPV X	1
**Tobacco non-users**			
	**hrHPV**		
		HPV 52	5
		HPV 16	4
		HPV 18, 39	2
		HPV 31	1
		HPV 39	1
		HPV 51	1
		HPV 66	1
		HPV 52, 44	1
		HPV 52, 54	1
		HPV 56, 74	1
		HPV 66, HPV X	1
		HPV 31, 52, 54	1
		HPV 31,16,18	1
		HPV 51, 53, 52	1
		HPV 52, 56, 74	1
		HPV 73, 39, 68	1
		HPV 16, 39, 58, 52	1
		HPV 18, 58, 39, 52	1
		HPV 73, 66, 39, 68	1
	**lrHPV**		
		HPV 74	3
		HPV 44	1
		HPV 44, HPV X	1
		HPV 54, 69/71, HPV X	1
		HPV 6	1
	**Unknown HPV type**		
		HPV X	15

HPV: human papillomavirus. hr: high-risk. lr: low-risk.

## DISCUSSION

Most of the study subjects (95.1%) were non-tobacco users, and only a small number (4.9%) were users. A positive HPV-DNA test was found in 4.35% (3/69) of the tobacco users and 3.93% (45/1145) in the non-users. The difference of 0.6% between these groups was considered significant because it exceeded the effect size (0.5%). However, analysis showed statistically insignificant result (unadjusted OR=1.19, p=0.778; 95% CI: 0.36–3.91; adjusted OR=1.18, p=0.786; 95% CI: 0.36–3.89). This statistically insignificant result may be due to an inadequate sample size. Power calculations showed that to have 80% power to analyze the association between tobacco use and HPV infection with minimum effect size 0.5%, type I error 5% and the proportion of positive HPV in tobacco users was assumed 4%, and the ratio of non-users and users was 20, thus a minimum 11884 tobacco users and 237678 non-users were required. However, the available data were only 1397 subjects.

A study by Lazcano-Ponce et al.^22^ in Mexico presented statistically similar results (OR=1.1; 95% CI: 0.6–2.0) with a total sample of 1340 subjects. The majority of the sample (76%) in their study had one lifetime sexual partner, and only 3% had multiple sexual partners during the previous years^22^. The study of Chatzistamatiou et al.^11^ observed that there was an association between smoking habits and high-risk HPV (hrHPV) infection in genitals in those aged 25–34 years (OR=2.3, p<0.001) while there was no association in other age groups.

A population-based study in Songkla and in Lampang, Thailand, conducted by Sukvirach et al.^23^ with 1741 samples, showed an inverse association between tobacco consumption and HPV infection. This study reported a lower risk (OR=0.45; 95% CI: 0.22– 0.92) of HPV-DNA infection in women with a history of tobacco smoking (19.8%). The smoking prevalence of women in Songkla, however, is low (6.1%), and in Lampang, it was reported higher in women aged >35 years (40.7%) than in younger women (2.3%). These data are similar to the prevalence of women smokers in Indonesia (4.8%) and the population in this study (5.68%). In the Sukvirach et al.^23^ study, a low participation rate was noticed because of limited screening (Pap smear) coverage in Thailand, and the proportion of women who refused to participate in the study was substantial. This study described the condition and obstacles of cervical cancer prevention in Asia, including in Indonesia.

Current smoking was a risk factor for squamous intraepithelial neoplasia if the HPV infection was persistent^24^. Higher HPV 16 and HPV 18 DNA load was also associated with current smokers (HPV16: p=0.03; HPV18: p=0.02), but not with former smokers (HPV16: p=0.35; HPV18: p=0.23)^10^. This study found that 2 of 3 tobacco users with positive HPV DNA were infected with HPV 16 or 18 as the predominant hrHPV group. In contrast, only 9 of 49 (18.4%) non-tobacco users were infected with HPV 16 or 18. Smoking can increase the oncogenic HPV infections duration and decrease the probability of oncogenic infections clearance^25^. Besides, HPV infection risk increased with the intensity of smoking, while the risk of former smokers (OR=0.95) is similar to non-smokers. Secondhand smoking and alcohol drinking women might be at high risk of hrHPV persistence, but active or secondhand smoking independently did not affect the risk^26^. However, the association between smoking and the persistence of HPV is still controversial and has not been fully clarified^27^.

Tobacco use, as one of the environmental/ exogenous factors, was not the only modifying factor contributing to the carcinogenesis of cervical cancer^28,29^. Although the specific mechanism of HPV infection-related tobacco use is unclear, it is presumed that tobacco has a harmful effect, particularly by direct carcinogenic process and local immuno-suppression in the cervix by reducing the number of Langerhans cells and CD4 lymphocytes, the local immune response in the uterine cervix. In addition, smoking can decrease natural killer cells’ activity, affecting the innate immune system^27^. However, the immunity status of the patients in this study could not be determined.

In the SDGs 2030, the UN includes tobacco control and poverty elimination^21^. Tobacco use and poverty have a marked correlation. It is believed that tobacco use has a detrimental impact on individuals, countries, and nations’ health and economy^21^. Moreover, higher poverty was also related to increased HPVassociated cancers^30^. Tobacco’s participation in increasing poverty has been confirmed, particularly in developing countries. Nevertheless, we observed that the number of smokers in Indonesia was much lower than in high-income countries. In this study, the total number of smoking women was 69/1397 (4.93%), consistent with the Indonesian data in 2018 (4.8%)^14^, while in SEAR, United States, and the world, these were 10.8%, 14.1%, and 6.1%, respectively^14,16,17^. In this study, poverty as a strong risk factor for cervical HPV infection could not be controlled for.

It has been recognized that the squamocolumnar junction (SCJ) is essential in VIA testing because an invisible SCJ can lead to false-negative results. However, in this study, we included subjects aged >50 years to show that postmenopausal women do not always have an invisible SCJ. In this study, women over 50 years were considered as post-menopause. The number of subjects over 50 years was 228, including only 79 subjects (35%) with an invisible SCJ, while 65% (149 subjects) still had a visible SCJ. In addition, all subjects with invisible SCJ were HPV-negative. These findings supported the conclusion that there was no probability of false-negative results in the invisible SCJ group of this study. Another study in the Indonesian population in 2016 found that 38.4% of post-menopausal subjects still had a visible SCJ ^31^.

The VIA is an excellent single screening tool with a sensitivity of over 90%^32^ and a low false-negative rate (3.21%)^33^. As a result, the false positive rate is high. In this study, each subject examination was performed by a trained general practitioner, midwife, or nurse, under the supervision of an expert and highly experienced gynecological oncologist to avoid bias due to false-positive and false-negative cases.

### Strengths and limitations

In this study, the distribution of positive HPV-DNA tests in the clinically normal uterine cervix was considered a rare study population. However, this study has some limitations. Firstly, although there was a slightly high proportion of HPV infection in tobacco users, it is not statistically significant. Secondly, we did not distinguish former versus active tobacco users and the severity or the amount of tobacco use which may confound the biology of HPV infection. Thirdly, the persistency of the HPV infection was not evaluated due to difficulty in contacting the subjects. Fourthly, sexual behavior, socioeconomic status as well as poverty, OCs use, Pap result, as well as tobacco consumption frequency as confounding factors were not controlled for due to limited information obtained in the medical records. Finally, the power of this study was not sufficient to prove statistically significance because of the insufficient sample size. Therefore, further research is still needed to improve the weaknesses in this study. Although this study has some limitations, we believe it can be generalized to the normal uterine cervix population in Indonesia.

## CONCLUSIONS

This study showed that there is no significant statistical association between tobacco use and HPV infection in normal uterine cervix. However, this result needs to be carefully interpreted. Moreover, the proportion of hrHPV infection was higher in tobacco users than in non-users. Tobacco use is still believed to increase the progression to cervical cancer in the current dysplasia of uterine cervix. From the SDGs perspective, cervical cancer is closely related to tobacco use and poverty. Tobacco contributes to increasing poverty, particularly in developing countries. We only discussed cervical cancer risk factors due to tobacco use. In addition, further research is still needed to improve the weaknesses in this study. Thus, further evaluation of other co-factor roles related to cervical cancer history is necessary to achieve the SDGs in 2030.

## Data Availability

The data supporting this research are available from the authors on reasonable request.
